# Molecular Pathways Regulating Macrophage Polarization: Implications for Atherosclerosis

**DOI:** 10.1007/s11883-012-0240-5

**Published:** 2012-03-11

**Authors:** Marten A. Hoeksema, J. Lauran Stöger, Menno P. J. de Winther

**Affiliations:** Department of Medical Biochemistry, Academic Medical Center, University of Amsterdam, Meibergdreef 15, 1105 AZ Amsterdam, The Netherlands

**Keywords:** Atherosclerosis, Cardiovascular disease, Coronary artery disease, Inflammation, Innate immunity, Macrophages, Foam cells, Gene regulation, Transcription factors, Epigenetics, Cytokines, Human pathology, Mouse models, Heterogeneity, Polarization, Microenvironment

## Abstract

Recent years have seen a tremendous development of our insight into the biology of atherosclerosis and its acute thrombotic manifestations. Inflammation now takes center stage among traditional risk factors as a decisive factor in cardiovascular risk. Consequently, its assessment and modulation have become key to clinical care and fundamental research alike. Plaque macrophages orchestrate many of the inflammatory processes that occur throughout atherogenesis. These cells are characteristically heterogeneous and adopt diverse activation states in response to micro-environmental triggers. In this review, macrophage-mediated inflammation in atherosclerosis sets the scene for a discussion of the gene regulatory mechanisms that facilitate and shape polarized macrophage phenotypes. When applicable, we consider these factors within the context of atherosclerosis and reflect on opportunities for future application.

## Introduction

Myocardial infarction and stroke remain among the leading causes of death and disease worldwide [1, 2]. To mitigate risk of these atherosclerotic complications, primary and secondary prevention strategies seek to correct aberrant blood cholesterol levels. Actively reducing low-density lipoprotein (LDL) cholesterol through lipid-modifying therapy (eg, statins) yields a proportional decrease in cardiovascular disease (CVD) risk [3]. However, there exists a considerable burden of residual risk, as current treatment strategies cannot prevent 75 % of major coronary events from occurring [4, 5]. Moreover, individuals afflicted by CVD are often times free of traditional risk factors [6], suggesting other dynamics contribute to plaque complication.

In this context, macrophage-mediated inflammation is paramount, contributing to atherosclerotic plaque initiation and progression through a variety of mechanisms [7]. We are developing a better understanding of the processes that regulate the induction and function of distinct macrophage subsets and their potential relevance in atherosclerosis. This review serves to highlight the cellular mediators that convert environmental cues to a heterogeneous array of functional macrophage phenotypes, thereby shaping inflammatory responses in health and disease.

## Inflammation and Atherosclerosis

Over the past two decades, the inflammatory hypothesis of atherothrombosis has gained an increasingly strong footing through multiple lines of supportive evidence. Overall, an increased systemic burden of inflammation prompts a higher CVD incidence, as is the case in chronic inflammatory conditions such as rheumatic arthritis [8] and systemic lupus erythematosus [9]. Various soluble mediators of the inflammatory response have been found to predict future cardiovascular risk in atherosclerotic patients (well-described in [10]). High-sensitivity C-reactive protein (hsCRP) has formed a focus point in this respect, as systemic concentrations of this acute-phase protein compared favorably with LDL cholesterol and blood pressure as CVD risk factors [11], and were specifically associated to plaque vulnerability [12, 13]. Building on post hoc analyses from several other large-scale studies (eg, CARE, PROVE-IT TIMI 22, AFCAPS/TexCAPS trials [14–16]), the JUPITER trial prospectively consolidated the correlation of hsCRP and cardiovascular outcome in a primary prevention setting [17]. Researchers observed that the clinical benefits of statin therapy were greatest when both LDL and hsCRP values were reduced, thus connecting both dyslipidemia and inflammation at the interface of CVD pathogenesis. Intriguingly, even with pre-existent LDL levels below the clinical cut-off point for treatment, persistent inflammation as measured by increased hsCRP levels puts patients at a higher than anticipated risk of CVD. In the AFCAPS/TexCAPS trial, these subjects responded strongly to treatment [16], indicating LDL burden is not a prerequisite to successful therapy. Apart from providing clinicians with valuable information for risk assessment, this finding proposes that an enhanced inflammatory state might in itself justify targeted therapy. Indeed, US and Canadian prevention guidelines have since embraced hsCRP measurements in the considerations for patients at intermediate risk. Moreover, a number of new trials, using either low-dose methotrexate (CIRT) or anti-IL-1β monoclonal antibodies (CANTOS) as anti-inflammatory treatment, are underway to address and possibly validate the hypothesis of inflammatory causality [18•, 19•]. These translational efforts could provide a major argument towards a more systematic implementation of anti-inflammatory therapy in our continuing battle to diminish residual cardiovascular risk.

Substantial experimental evidence complements the broad clinical involvement of inflammation in CVD outlined above. Now most agree that systemic risk factors interact with many cell types (both those intrinsic to the vasculature and immune cells attracted from the circulation) to drive plaque development. Particularly, monocyte-derived macrophages are considered critical participants in the atherogenic process, as they secrete pro-inflammatory cytokines and other mediators that affect lesion progression and stability. Consequently, many experimental studies have successfully targeted the abundance of monocytes/macrophages and their soluble repertoire in atherosclerosis as a means of prevention. For instance, atherosclerotic plaque formation was virtually abolished in hyperlipidemic mice lacking the macrophage-colony stimulating factor (M-CSF) gene, which exhibit impaired monocyte development and subsequent differentiation to macrophages [20, 21]. Other scientific efforts involved the abrogation of chemokine-dependent monocyte recruitment to the plaque [22], in addition to a wealth of studies addressing the various cytokines produced by macrophages and other cells (reviewed in [23]). Although not cell-specific, these data still offer valuable insight into how macrophages contribute to nascent lesions. Macrophage apoptosis is another important feature seen during atherosclerosis development. In early lesions, macrophage apoptosis and plaque size exist in an inverse relationship [24], whereas in later stages this process contributes to the plaque’s lipid core [25]. This ambiguity appears to be mediated by a process termed “efferocytosis” [26]. Combined with proof linking plaque macrophages to matrix metalloproteinase (MMP)-dependent collagen breakdown [27, 28], it is evident that these cells modulate inflammatory mechanisms to determine plaque susceptibility to rupture and clinical atherothrombosis. Associative imaging studies lend weight to this notion by demonstrating that vascular uptake of fluorodeoxyglucose (^18^F-FDG) correlates to plaque macrophage load and general inflammatory burden and can thereby assist in the prediction of cardiovascular events [29, 30].

## Phenotypic Differences Between Macrophage Subsets

Cultured and tissue macrophages both exhibit pronounced heterogeneity, as was recognized early on [31]. Skewing of macrophages toward distinct polarization programs occurs in response to various environmental cues and has inspired extensive research into their significance in pathophysiology. Reflecting the Th1 and Th2 nomenclature in T-cells, polarized macrophage subsets were originally referred to as classically (M1) or alternatively activated (M2) [32]. The latter group was subsequently divided into M2a, M2b, M2c and tumor-associated macrophages (TAMs) to distinguish between inducing stimuli [33]. Later on, Mosser and Edwards [34] reassigned these macrophage subsets to three different classes: classically activated macrophages (CAMs, corresponding to M1), alternative activated macrophages (AAMs, also referred to as wound healing macrophages and analogous to M2a), and regulatory macrophages (RMs, consistent with M2b/c). These classes are best considered a continuum of functional states that encompasses a broad range of macrophage phenotypes with interchangeable characteristics.

CAMs are typically induced by the Th1-cytokine interferon-γ (IFNγ), possibly followed by activation with a Toll-like Receptor (TLR) ligand, such as lipopolysaccharides (LPS). IFNγ (originally termed macrophage-activating factor) prepares macrophages for a pro-inflammatory environment, giving rise to a potent inflammatory response upon microbial challenge. The resulting phenotype characteristically displays high interleukin-12 (IL-12) and low IL-10 production, combined with enhanced microbicidal effector functions through the induction of NADPH-oxidases and inducible nitric oxide synthase (iNOS). Therefore, these macrophages become very efficient in killing bacteria, viruses, parasites, and fungi. Their continuous induction and sustained activation, however, will cause tissue damage [35].

The Th2 cytokines IL-4 and IL-13 are produced by granulocytes, mast cells, and Th2 cells during injury and infection and generate AAMs [36, 37]. Opposite to CAMs, AAMs dampen inflammatory responses through an IL-12^low^ and IL-10^high^ expression profile. Furthermore, AAMs promote tissue repair and fibrosis through increased arginase-1-dependent production of the collagen precursors ornithine and proline [38]. Besides arginase-1, other markers for AAMs include Ym1, Fizz1, and the mannose receptor (MR).

Finally, regulatory macrophages (RMs) are induced in response to a wide range of stimuli, including immune complexes, prostaglandins, G-protein coupled receptor ligands, glucocorticoids, and uptake of apoptotic cells. However, a key cytokine in the induction of RMs is the anti-inflammatory, atheroprotective cytokine IL-10. The main task of RMs is to suppress and control immune responses by producing high levels of IL-10 and thereby contribute to the resolution of inflammatory responses [34, 39].

## Macrophage Differentiation and Polarization

The current framework of macrophage subsets is very well characterized in vitro but less so in in vivo settings. Here, a greater variety of external challenges elicit macrophage phenotypes that are considerably less adherent to the constraints of the existing paradigm. During monocyte-to-macrophage differentiation and subsequent macrophage activation, the collective imprint of these environmental factors shapes the macrophage phenotype. An intuitive overview of these sequences was recently described by Gordon and Martinez [40].

First, monocyte recruitment from the circulation will be followed by differentiation to mature tissue macrophages. This transition is primarily mediated by the growth factors M-CSF and granulocyte-macrophage colony-stimulating factor (GM-CSF) that influence the inflammatory potential of the resulting macrophage. Maturation with M-CSF was observed to lead to a more anti-inflammatory (IL-10^high^IL-12^low^) phenotype, whereas GM-CSF-induced differentiation gave rise to a macrophage population with pro-inflammatory (IL-10^low^IL-12^high^) characteristics [41, 42].

The phenotype that ensues from the subsequent priming stage hinges on the local balance of cytokines and chemokines in the newly recruited macrophage’s environment. Priming with cytokine stimuli will affect the inflammatory potential of macrophages and their response to other stimuli. In addition to the previously described cytokine stimuli (IFNγ, IL-4/IL-13, and IL-10 for CAMs, AAMs, and RM, respectively), chemokines (eg, CXCL4) [43] and other plaque constituents (oxidized lipoproteins) [44] were also shown to induce unique macrophage phenotypes with distinctive characteristics.

Upon activation by Toll-like or analogous receptor stimuli, the macrophage will undergo functional maturation that results in a rapid induction of anti-microbial pathways for fast killing and clearance of pathogens. Finally, when the macrophage has fulfilled and survived its inflammatory task, it will undergo deactivation. In this final phase, the RM phenotype and its mediators transforming growth factor-β (TGF-β), IL-10, and lipoxins, are key contributors to the resolution of inflammation and tissue repair [40].

## Macrophage Polarization and Disease

Macrophage subtypes have the capacity to switch from one phenotype to another as stimuli from the micro-environment change. Recently, such plasticity was elegantly demonstrated in a murine kidney ischemia-reperfusion model [45•]. Whereas initially pro-inflammatory CAMs were recruited to the kidney, later time points saw previously attracted macrophages switch to an MR^+^-AAM phenotype, signaling the resolution of inflammation and repair. Similar reports were made with regard to other disease states, as for instance in metabolic disease. Adipose tissue macrophages in lean mice resemble an AAM phenotype that supports adipocyte function and insulin sensitivity [46]. Obesity, however, switches the macrophage balance to a CAM phenotype as the result of inflammasome activation by various danger signals from adipocytes, thereby promoting inflammation and insulin resistance [47, 48]. In analogy to this dichotomy, differential macrophage function has been reported to influence the course of various other conditions, such as cancer and infectious disease [34, 49].

**Fig. 1:**
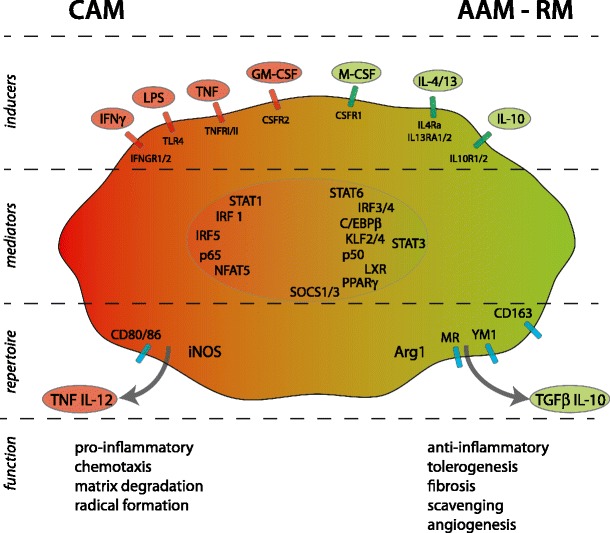
Molecular regulation of macrophage subsets. From the top down are indicated key external factors that drive macrophage polarization either to the classically activated macrophage (CAM) direction *(left side*) or to the alternative activated macrophages/regulatory macrophages (AAM/RM) direction (*right side*), the receptors involved, transcription factors that regulate CAM or AAM/RM, and some key markers that characterize the subsets. At the bottom, implications of the subsets for atherosclerosis are indicated, which are discussed in more detail in two recent reviews [49, 56]

Likewise, in atherosclerosis, evidence implies that the balance of macrophage polarization is crucial in determining plaque outcome [49–51]. Following the progression of atherosclerosis over time, it was shown that plaque macrophages in early lesions express arginase-1 (indicative of AAMs), whereas at later time points the expression of arginase-2 (referred to as a CAM marker) predominated in the plaque [52]. More recently, phenotypic switching in atherosclerosis was described upon induction of disease regression [53••]. Here, plaque macrophages restrained pro-inflammatory marker expression (eg, monocyte chemotactic protein-1 and tumor necrosis factor) and enhanced that of AAM markers such as arginase-1, MR, and CD163 upon normalization of the plasma lipid profile. However, the order of events in this model still requires clarification (ie, does phenotypic switching actively contribute to plaque regression or is it merely a consequence thereof). In human subjects too, it was shown that symptomatic atherosclerotic disease of the carotid arteries is associated with a CAM profile, characterized by high pro-inflammatory cytokine expression [54]. Moreover, enrichment of CD11c^+^-CAMs over MR^+^-AAMs characterizes the epicardial adipose tissue from patients with manifest coronary artery disease [55]. These reports are consistent with the current hypothesis that CAMs propagate plaque vulnerability through their inflammatory potential, whereas their AAM counterparts are likely to benefit plaque stability and regression through a repertoire of repair and fibrotic functions. From this perspective, Fig. 1 provides a schematic overview of the inducers, transducers, and effector functions of these macrophage populations in atherosclerosis development.

Although murine macrophage subtypes are well characterized, the translation to human pathophysiology is not straightforward. Where polarized macrophage subsets in mice can be easily distinguished based on their arginine-metabolizing enzyme expression (i.e. iNOS and arginase-1 for CAMs and AAMs respectively), in vitro polarized human macrophages do not express arginase-1 and fail to produce NO in amounts comparable to that of mouse macrophages [57]. In addition, markers like Ym1 and Fizz1, which are excellent for AAM detection in mice, are not expressed in humans [35]. Circumventing these issues through identification of generally more favorable markers of human tissue macrophages will prove an important step in translational atherosclerosis research.

## Transcription Factors in Macrophage Subsets

To achieve expression of the aforementioned surface marker and cytokine profiles, different macrophage subsets are known to employ distinct signaling pathways (Fig. 1). These cascades usually activate certain transcription factors that effectively induce expression of archetypal genes, thereby accounting for the translation of external polarizing cues into a specific subtype [58]. Clearly, this designates transcription factors as important players in macrophage polarization that may represent an attractive alternative to surface markers for discerning macrophage populations in vivo. Moreover, these pathways may eventually yield suitable targets for the inhibition, depletion, or stimulation of a particular phenotype. An overview of transcription factors associated to macrophage heterogeneity and their functional relevance in experimental or human atherosclerotic lesions, as described below, can be found in Table 1.

**Table 1:** The role of transcription factors in macrophage polarization and atherosclerosis

Gene	Linked?	Model	Findings	Refs
Classically activated macrophage				
STAT1	Yes	ApoE^-/-^+LDLR^-/-^; BMT	Hematopoietic STAT1 deficiency leads to decreased plaque sizes	[59–61]
NF-κB p65	Yes	Human plaques	The presence of activated NF-κB p65 in human atherosclerotic plaques was demonstrated	[64]
IRF1	Yes	Human lymphocytes	IRF-1 expression was increased in lymphocytes from patients with acute coronary syndrome	[72, 74]
IRF5	No	N/A	N/A	[70, 71]
NFAT5	Yes	ApoE^-/-^	NFAT5 expression was highly expressed in murine atherosclerotic lesions	[82, 83]
SOCS1^a^	Yes	Human plaques	SOCS1 and SOCS3 are highly expressed by vascular smooth muscle cells and macrophages in inflammatory regions	[84, 85]
SOCS3	Yes	ApoE^-/-^	SOCS3 targeting oligonucleotides exacerbated the atherosclerotic process	[85, 86]
Regulatory macrophage				
STAT3	Yes	LDLR^-/-^; adenovirus	Adenovirus expression of human STAT3 lowers aortic inflammatory cell infiltration	[87, 88]
Alternatively activated macrophage				
STAT6	No	N/A	N/A	[62]
NF-κB p50	Yes	LDLR^-/-^; BMT	p50 deletion in bone marrow cells leads to more inflammatory and smaller lesions	[67, 68]
IRF3	No	N/A	N/A	[41]
IRF4	No	N/A	N/A	[69]
PPARγ	Yes	Human monocytes; IHC human plaques	PPARγ activation protects against atherosclerotic plaque formation in humans	[75, 76, 81•]
LXRα	Yes	LDLR^-/-^; lentivector and human plaques	Macrophage LXRα gene therapy ameliorates atherosclerosis in LDLR^-/-^ mice and LXRα was found increased during regression. In contrast, human AAM plaque macrophages were shown to have decreased levels of LXRα.	[77, 79, 81•]
Cebpb	No	N/A	N/A	[89]
KLF2	Yes	KLF2^+/-^ ApoE^-/-^	Hemizygous KLF2 deficiency increased atherosclerosis in apoE^-/-^ mice	[90, 91]
KLF4	No	N/A	N/A	[92]
SOCS1^a^	Yes	Human plaques	SOCS1 and SOCS3 are highly expressed by vascular smooth muscle cells and macrophages in inflammatory regions	[84, 85]

^a^ Involved in both AAM as CAM signaling

*ApoE*—apolipoprotein E; *BMT*—bone marrow transplantation; *Cebpb*—CCAAT/enhancer-binding protein beta; *IHC*—immunohistochemistry; *IRF*— Interferon regulatory factors; *KLF*—Krüppel-like factor; *LDLR*—low-density lipoprotein receptor; *LXR*—liver X receptor; *N*/*A*— not applicable; *NFAT*—nuclear factor of activated T cells; *PPAR*—peroxisome proliferator-activated receptor; *SOCS*—suppressor of cytokine signaling proteins; *STAT*— signal transducers and activators of transcription

### STAT Signaling

Signal transducers and activators of transcription (STATs) are crucial transcription factors in the determination of macrophage phenotype. IFNγ signaling will lead to downstream phosphorylation and activation of STAT1. Macrophages lacking this mediator are unable to express a full CAM phenotype, as they fail to produce nitric oxide (NO) and have diminished expression of IL-12 and major histocompatibility complex (MHC) class II [59]. In murine atherosclerosis, macrophage STAT1 deficiency leads to attenuated atherosclerosis, resulting from a decrease in macrophage lipid accumulation, macrophage apoptosis, and plaque necrosis [60, 61]. IL-4 and IL-13, inducers of the AAM phenotype, also signal via JAK-STAT pathways. Both cytokines lead to the activation of STAT6. STAT6 signaling was shown to be necessary for the expression of many AAM markers, like arginase-1 [62], but has not been directly linked to atherosclerosis development.

### NF-κB Signaling

Nuclear factor κB (NF-κB) is a key regulator of the initiation and resolution of inflammation [63]. NF-κB activation, more specifically that of its subunit p65, is a hallmark of classical macrophage activation and occurs in both endothelial cells, smooth muscle cells, and macrophages in human atherosclerotic lesions [64]. However, its activation is subject to multiple levels of (inhibitory) regulation that fine-tune macrophage function. Interestingly, attempts to inhibit NF-κB activity by deleting inhibitor of NF-κB kinase (IKK) 2, the kinase that activates NF-κB, yielded unexpected outcomes. IKK2 was shown to inhibit STAT1 signaling in macrophages, thereby affecting IL-12, iNOS, and MHC class II expression [65]. Therefore, IKK2 deletion did not diminish polarization towards the CAM phenotype, but instead encouraged certain CAM characteristics through enhanced STAT1 activity. As such, macrophage-specific deletion of IKK2 in atherosclerosis resulted in reduced NF-κB activity but an increased atherosclerotic plaque size [66].

Conversely, the p50 subunit of NF-κB was recently identified as a key regulator of the AAM phenotype both in vitro as in vivo [67]. In line, it was shown that targeted deletion of the p50 subunit in macrophages in atherosclerosis led to more inflammatory lesions. Surprisingly, these lesions were smaller in size, which was linked to a reduced uptake of oxidized LDL in activated p50-deficient macrophages [68].

### IRF Signaling

Interferon regulatory factors (IRFs) are important mediators of macrophage polarization and several family members have been reported in relation to a specific phenotype. IRF4 was shown to be a key transcription factor controlling AAM polarization and its associated gene expression profile [69]. High expression of IRF5 was observed in CAMs and subsequently described as a central mediator in TLR signaling pathways. Here, it will directly activate the transcription of pro-inflammatory genes and repress anti-inflammatory IL-10 expression [70, 71]. Likewise, IRF1 was shown to cooperate with NF-κB in the induction of several pro-inflammatory cytokines [72]. Moreover, by antagonizing IRF4 function [73], IRF1 can also be classified as an important mediator in CAM signaling. Although the role of IRFs in atherosclerosis is still to be determined, lymphocytes from patients with acute coronary syndromes display increased IRF-1 expression, linking this transcription factor to cardiovascular risk [74].

### PPAR and LXR Signaling

Another transcription factor found to be crucial for murine AAM polarization is peroxisome proliferator-activated receptor (PPAR) γ, a nuclear receptor [75]. Also in human monocytes, it was shown that PPARγ activation skews towards an AAM phenotype and conveys important functional differences [76]. A closely related nuclear receptor is liver X receptor (LXR) α, which was found to be of similar importance in AAM polarization. Besides regulating lipid metabolism and transport, LXRα enhances arginase-1 expression and suppresses inflammatory signaling in macrophages [77]. Both of these transcription factors fulfill additional protective roles in experimental atherosclerosis [78–80], mainly by reducing intra-cellular accumulation of oxidized LDL [76, 81•]. Therefore, PPARγ and LXRα instill macrophages with powerful anti-inflammatory and metabolic functionalities.

## Epigenetic Control of Macrophage Polarization Through Histone Modifications

In addition to transcriptional control, epigenetic regulation is essential for a properly directed expression of target genes. Without modifying the actual genetic code, epigenetic mechanisms affect DNA accessibility to the transcriptional apparatus to alter gene expression and even mRNA degradation by microRNAs. Remarkably, the resulting gene expression patterns can be passed down to daughter cells upon cell division or even trans-generationally. Epigenetic regulation can be achieved through DNA methylation (generally considered a relatively static process), as well as through applying certain modification to histones, such as acetylation or methylation. These marks can be changed very dynamically in response to many environmental stimuli.

In macrophages, several epigenetic mediators now are identified as key players in macrophage function and polarization [93]. For instance, Jmjd3, a JmjC family histone demethylase that erases lysine 27 trimethylation marks on histone 3, has been shown to specifically bind transcription start sites of promoters of NF-κB dependent genes. This would suggest that Jmjd3 provides additional control of NF-κB-dependent gene expression, although most pro-inflammatory genes were expressed independently of Jmjd3 [94, 95]. In addition, it has been described that Jmjd3 plays an essential role in polarization. In response to AAM polarization, Jmjd3 can induce the expression of IRF4, resulting in the transcription of key AAM marker genes [62, 69]. Recently, the histone deacetylase 3 (HDAC3) was also found to mediate macrophage polarization, as HDAC3-deficient macrophages were shown to be hyperresponsive to polarization with IL-4 [96]. Thus, alternative activation of macrophages can be controlled at an epigenetic level by targeting HDAC3 and Jmjd3, suggesting that epigenetic mediators might evolve to become promising immunomodulatory targets. This notion was recently substantiated by the finding that the bromodomain and extra terminal domain family of proteins-inhibitor (I-BET) is able to disrupt chromatin complexes responsible for the expression of key inflammatory genes during macrophage activation and could thereby protect against endotoxic shock and sepsis [97•]. This indeed very nicely demonstrates that synthetic compounds specifically targeting epigenetic mechanisms can act as excellent therapeutic agents for immunomodulation. As future studies further unravel the epigenomic landscape of macrophage subtypes, we will definitely see the development of other immunomodulatory compounds that target epigenetic regulation of macrophages. Moreover, in experimental atherosclerosis, it was shown that cell-specific histone methylation modifications and expression of accompanying lysine methyltransferases occur in carotid arteries. More specifically, differences in histone methylation modifications in vascular endothelial and smooth muscle cells were described between the offspring of hypercholesterolemic and normocholesterolemic mothers [98]. As histone modifications are readily altered in response to environmental stimuli, they can provide an attractive explanation how diet and lifestyle may contribute to atherosclerosis susceptibility [99].

## Conclusions

Amongst a variety of atherogenic properties, macrophages direct and amplify the inflammatory response in atherosclerosis and thereby contribute to lesion initiation, progression, and clinical manifestation. The plaque macrophage phenotype is determined by a plethora of micro-environmental stimuli encountered during the successive phases of macrophage development. These factors can trigger activation of subtype-specific signaling pathways and downstream transcription factors, which are crucial mediators of macrophage polarization. Additionally, epigenetic modifiers comprise a relatively novel level of transcriptional regulation that has been shown to fine-tune the macrophage phenotype. Together, these molecular effectors may govern the critical balance between distinct macrophage subsets in atherosclerosis. Such modulation could in turn be expected to either propagate plaque progression or to confer atheroprotective mechanisms. It therefore remains crucial to improve our understanding of macrophage heterogeneity in atherosclerosis and identify the subsets most suitable for intervention.

Key areas for careful consideration in that light include the longstanding need for better, more specific markers that characterize human macrophage subsets. However, interspecies differences in atherosclerotic pathogenesis and macrophage marker expression currently prevent us from taking the next step in translational research [100]. Paradoxically, additional work with animal models will prove indispensable to addressing these challenges, because these approaches allow for specific deletion of transcriptional regulators involved in macrophage polarization in experimental atherosclerosis. These endeavors will provide us with great mechanical insight. Subsequently, by isolating those mediators important to murine polarization and ablating them in human macrophages, we can identify new polarization markers or even a polarization-specific gene profile for macrophage subsets. Alternatively, the activity of polarizing transcription factors could be used as a measure of the inflammatory state of atherosclerotic lesions. Thereby, gene regulation pathways may be valuable for diagnosis and individual risk assessment, when combined with lipid profiles and CRP-levels. In the future, one might even think of monitoring the activity of these pathways to evaluate the effects of therapeutic intervention.

As such, the exploration of transcription factors and epigenetic modifiers that uniquely shape the macrophage phenotype has the ability to advance our insight into the macrophage polarization paradigm considerably. This upholds its potential relevance for integration into the diagnostic and therapeutic algorithms of human atherosclerosis, as described above. Ultimately, we expect the greatest benefit from cell type-specific strategies that allow us to gently shift the plaque macrophage towards a desirable, atheroprotective phenotype, possibly by targeting the appropriate signaling pathways.
